# Clinical Diagnosis and Treatment of Scurvy in a Young Adult With Poor Dietary Habits: A Case Report and Literature Review

**DOI:** 10.1155/2024/2779253

**Published:** 2024-10-25

**Authors:** Lara Saeid, Moatasem Hussein Al-Janabi, Fouz Hassan

**Affiliations:** ^1^Department of Hematology, Tishreen University Hospital, Lattakia, Syria; ^2^Department of Pathology, Cancer Research Center, Tishreen University Hospital, Lattakia, Syria; ^3^Department of Dermatology, Tishreen University Hospital, Lattakia, Syria

**Keywords:** ascorbic acid, diagnosis, poor diets, scurvy, treatment, vitamin C deficiency

## Abstract

Scurvy, once prevalent among sailors, is now rare due to improved access to fresh fruits and vegetables yet persists in individuals with poor dietary habits. We report a case of a 35-year-old male presenting with month-long, nonpainful, nonitchy lower extremity lesions. A dermatological examination revealed follicular hyperkeratosis, perifollicular bleeding, corkscrew hairs, bleeding gums, and hemorrhagic purpura. Despite unavailable plasma vitamin C testing, scurvy was diagnosed based on clinical features and dietary history. Treatment with vitamin C resulted in significant improvement within 10 days. Inadequate intake of fruits and vegetables causes vitamin C deficiency, impairing collagen synthesis and leading to typical scurvy symptoms. This case underscores the importance of considering scurvy in patients with compatible symptoms, focusing on clinical diagnosis and treatment response when testing is unavailable. Management includes vitamin C supplementation and dietary changes, emphasizing healthcare providers' role in promoting sufficient fruit and vegetable consumptions to prevent nutritional deficiencies.

## 1. Introduction

Scurvy, caused by a deficiency of vitamin C, predominantly arises from inadequate dietary intake of vitamin C-rich foods such as citrus fruits and tomatoes [1]. Although rare in modern-developed societies, it persists in individuals with poor diets, particularly those with restricted access to fresh produce or specific dietary restrictions [2, 3]. It can also be seen in individuals with psychiatric disorders or gastrointestinal conditions that limit nutrient absorption [4, 5]. This case report presents a 35-year-old male with characteristic symptoms of scurvy, highlighting the importance of considering this diagnosis in patients with poor dietary habits, even in the absence of socioeconomic risk factors.

## 2. Case Presentation

A 35-year-old male patient visited the dermatology clinic with a history of lesions on his lower extremities, first noticed a month before his visit. The lesions were neither painful nor itchy.

The patient, a nonsmoker and nonalcoholic, reported no chronic diseases in his medical history. However, he did have a history of esophageal irritation, triggered specifically by certain foods including citrus fruits. Despite these dietary restrictions, a recent gastroenterology evaluation showed no signs of gastrointestinal disease, and no medications were prescribed.

**Figure 1:**
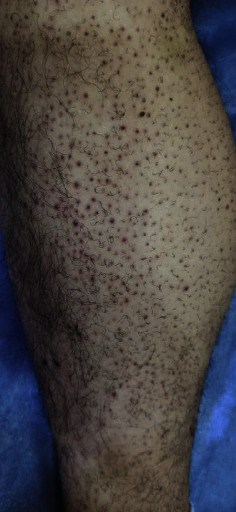
A clinical image of the patient shows follicular hyperkeratosis, perifollicular bleeding, and corkscrew hairs on the lower extremities.

**Figure 2:**
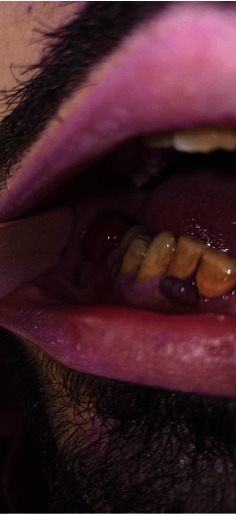
A clinical image of the oral cavity reveals multiple hematomas, along with hemorrhagic purpura on the palate.

Additionally, the patient reported systemic symptoms including headaches and fever. During the clinical examination, dermatological findings included follicular hyperkeratosis, perifollicular bleeding, and corkscrew hairs on the lower extremities (Figure 1). Examination of the oral cavity revealed easily bleeding gums following minor trauma and multiple hematomas, along with hemorrhagic purpura on the palate (Figure 2).

Laboratory tests were largely unremarkable with most values within normal limits, except for an elevated erythrocyte sedimentation rate (ESR) of 41 mm/hr. A chest x-ray revealed no abnormalities.

**Figure 3:**
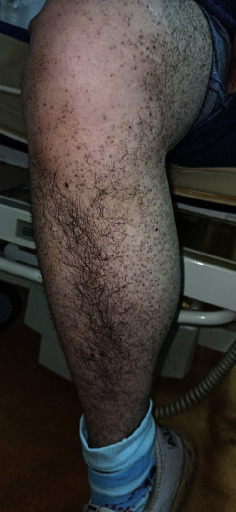
A clinical image of the skin lesions after treatment shows significant improvement.

**Figure 4:**
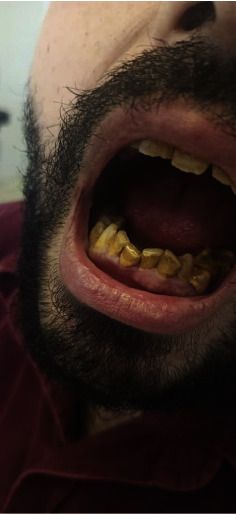
A clinical image of the oral cavity after treatment shows no evidence of hematomas.

Based on the clinical findings, a diagnosis of scurvy was made. Plasma vitamin C concertation was not performed because it was not available in our hospital. Treatment was initiated with vitamin C and administered at a dosage of 500 mg twice daily. Within 10 days, the patient showed remarkable recovery, with significant improvement in both systemic and dermatological symptoms (Figures 3 and 4).

## 3. Discussion

Scurvy, historically linked to vitamin C deprivation, remains a relevant concern for individuals with poor diets, even in developed countries [2, 6]. Our patient presented with classic dermatological and mucocutaneous manifestations such as corkscrew hairs and gum bleeding, hallmark signs of vitamin C deficiency. His avoidance of vitamin C-rich foods due to esophageal irritation led to chronic deficiency. This case demonstrates that scurvy can be clinically diagnosed in the absence of plasma vitamin C testing, with a positive response to supplementation serving as confirmation.

Typically, diagnosis of scurvy relies on plasma or serum vitamin C level testing (normal range: 0.4–2 mg/dL). However, when such testing is unavailable, as in our case, a clinical diagnosis based on symptoms and dietary history, along with rapid improvement after vitamin C supplementation, is sufficient.

A review of the literature reveals similar patterns among patients diagnosed with scurvy (Table 1), showing that while scurvy is uncommon in developed countries, it can still occur in diverse demographics, particularly those with dietary deficiencies or malabsorption syndromes. In this case, the lack of fresh produce in the patient's diet resulted in impaired collagen synthesis, which led to the characteristic symptoms of scurvy [1, 2, 6, 7]. Inadequate collagen production affects the integrity of connective tissues, leading to mucocutaneous bleeding, joint pain, and impaired wound healing [3].

It is important to note that scurvy can also present with neuropsychiatric symptoms, such as depression, cognitive disturbances, and mood changes. Although these were not observed in our patient, they are well-documented in the literature [8].

Treatment for scurvy involves replenishing vitamin C levels through oral or intravenous supplementation, combined with dietary modifications to ensure adequate intake of vitamin C-rich foods [1, 4]. In cases of malabsorption, oral supplementation may be ineffective, necessitating intravenous administration [9]. In our patient, high-dose oral vitamin C supplementation led to rapid improvement in symptoms.

While scurvy is commonly associated with populations at risk of malnutrition, such as the elderly or those with limited access to fresh foods [3], this case highlights that scurvy can also occur in young, otherwise healthy individuals with poor dietary habits. It underscores the importance for healthcare providers to remain vigilant for scurvy in patients with poor diets, regardless of their age or overall health status. Additionally, this case emphasizes the critical role of patient education in promoting a balanced diet rich in fruits and vegetables to prevent nutritional deficiencies.

## 4. Conclusion

This report highlights the importance of considering scurvy in patients with compatible clinical features, even when testing is unavailable. Rapid treatment with vitamin C can lead to significant improvement, emphasizing the crucial role of a balanced diet in preventing deficiencies.

## Figures and Tables

**Table 1:** Comparison of cases of scurvy in the literature since 2012: patient demographics, presenting symptoms, diagnosis methods, treatment, and outcomes.

Study (Author, year)	Patient demographics	Presenting symptoms	Diagnosis method	Treatment	Outcomes
Premkumar et al. 2024 [3]	4-year-old child, malnutrition	Difficulty walking and sitting	Vitamin C plasma levels	Vitamin C supplementation	
Lu et al. 2023 [1]	25-year-old male, poor diet	Pain, weight loss, mild swelling	Clinical examination, blood tests.	Vitamin C, folate, and mecobalamin supplementation	Improvement in 4 weeks
Thomas and Burtson 2021 [4]	69-year-old female, medical history	Ecchymoses, poor dental situation, arrived with unstable BP	Vitamin C plasma levels	Vitamin C supplementation	Symptoms resolved in 2 weeks
Wijkmans and Talsma 2016 [2]	35-year-old male, malnutrition	Extensive bruising on the right leg	Physical exam, vitamin C plasma levels	Supplements	Patient reported improvement
Noordin et al. 2012 [10]	4-and-a-half-year-old boy	Swollen joints, inability to walk	Blood tests, X-rays	Supplements	Symptoms resolved in 4–6 weeks
Our case	35-year-old male, poor diet	Cutaneous lesions, corkscrew hair, easy bleeding	Clinical examination	Vitamin C supplementation	Symptoms resolved in 10 days

## Data Availability

Data sharing is not applicable to this article.
